# Leptin and Ghrelin in Excessive Gestational Weight Gain—Association between Mothers and Offspring

**DOI:** 10.3390/ijms20102398

**Published:** 2019-05-15

**Authors:** Jolanta Patro-Małysza, Marcin Trojnar, Katarzyna E. Skórzyńska-Dziduszko, Żaneta Kimber-Trojnar, Dorota Darmochwał-Kolarz, Monika Czuba, Bożena Leszczyńska-Gorzelak

**Affiliations:** 1 Chair and Department of Obstetrics and Perinatology, Medical University of Lublin, Lublin 20-090, Poland; zkimber@poczta.onet.pl (Ż.K.-T.); monikaczuba77@o2.pl (M.C.); b.leszczynska@umlub.pl (B.L.-G.); 2 Chair and Department of Internal Medicine, Medical University of Lublin, Lublin 20-081, Poland; marcin.trojnar@umlub.pl; 3 Chair and Department of Human Physiology, Medical University of Lublin, Lublin 20-080, Poland; katarzyna.skorzynska-dziduszko@umlub.pl; 4 Department of Gynecology and Obstetrics, Institute of Clinical and Experimental Medicine, Medical Faculty, University of Rzeszow, Rzeszów 35-959, Poland; ddarmochwal@ur.edu.pl

**Keywords:** excessive gestational weight gain, neonatal anthropometry, leptin, ghrelin

## Abstract

Two-thirds of pregnant women exceed gestational weight gain recommendations. Excessive gestational weight gain (EGWG) appears to be associated with offspring’s complications induced by mechanisms that are still unclear. The aim of this study was to investigate whether umbilical cord leptin (UCL) and ghrelin (UCG) concentrations are altered in full-term neonates born to EGWG mothers and whether neonatal anthropometric measurements correlate with UCL and UCG levels and maternal serum ghrelin and leptin as well as urine ghrelin concentrations. The study subjects were divided into two groups, 28 healthy controls and 38 patients with EGWG. Lower UCL and UCG levels were observed in neonates born to healthy mothers but only in male newborns. In the control group UCG concentrations correlated positively with neonatal birth weight, body length and head circumference. In the control group maternal serum ghrelin levels correlated negatively with neonatal birth weight, body length and head circumference as well as positively with chest circumference. In the EGWG group UCG concentrations correlated negatively with neonatal birth weight and birth body length. UCL correlated positively with birth body length in EGWG group and negatively with head circumference in the control group. In conclusion, EGWG is associated with disturbances in UCL and UCG concentrations.

## 1. Introduction

According to the current state of knowledge, excessive gestational weight gain (EGWG) as well as pre-pregnancy obesity appear to be associated with long-term sequelae in the offspring. Prenatal life may be of importance as a ‘critical period’ since it is the time when the risk of development and persistence of dyslipidemia, overweight, obesity, impairments in cognition, neuropsychiatric disorders, cardiovascular diseases and metabolic syndrome in the future life of the offspring is increased [1,2,3,4]. Maternal hyperinsulinemia, hyperleptinemia and inflammation are associated with excessive nutrient transport at the placental level. EGWG, which is usually due to improper nutrition during the pregnancy period, has been regarded as a potentially modifiable, independent risk factor for excessive offspring growth and serious metabolic disorders [3,5].

Gestational weight guidelines of the Institute of Medicine (IOM) [6] provide ranges of recommended weight gain for specific pre-pregnancy body mass index (BMI) categories in relation to the least risk of adverse perinatal outcomes. In order to minimize the risk of maternal and infant complications it has been suggested that weight gain in pregnancy should not exceed 11.5–16, 7–11.5 and 5–9 kg in women with normal pre-pregnancy BMI, overweight and obese subjects, respectively [6]. More than two-thirds of pregnant women exceed gestational weight gain recommendations of the IOM [7].

Leptin, an adipocyte-derived satiety factor, is known to reduce food intake and raise/boost energy expenditure. However, due to the increase of leptin levels in the maternal blood in the second half of pregnancy, leptin resistance develops [8,9], which, especially in late pregnancy, is thought to be mediated by the placental secretion hormones, i.e., prolactin and placental lactogen family of the molecules [10,11].

Ghrelin, an acylated peptide hormone, plays a crucial role in energy homeostasis and it has been demonstrated to stimulate fetal development by binding to the growth hormone receptors [12]. Ghrelin is also an important factor linking the central nervous system with the peripheral tissues that regulate energy homeostasis and lipid metabolism [12]. A physiological increase of maternal and fetal ghrelin levels has been observed during pregnancy in mammals (including humans) [13,14]. In light of this, it seems plausible that ghrelin is likely to be one of many peptides engaged in the process of fertilization as well as in preimplantation embryo development and implantation; intragestational ghrelin participates in reproductive fetal programming [15]. Ghrelin levels can be affected by multiple factors, including diet composition, exercise, environment and lifestyle [12]. 

Disrupted leptin and ghrelin secretion homeostasis may result in production of improper hypothalamic signals, thereby bringing a feeling of hunger, which will lead to excessive food consumption and lipogenesis. All of this has been displayed in both animal and human models [11].

Even though numerous studies have performed leptin and ghrelin blood concentration measurements in both healthy and unhealthy individuals [11,12,13], the data on the relative role of maternal and fetal leptin and ghrelin in the fetal growth are still patchy. The aim of this study was to investigate whether the umbilical cord leptin and ghrelin concentrations are altered in full-term neonates born to EGWG mothers and whether neonatal anthropometric measurements correlate with the umbilical cord blood and maternal serum leptin and ghrelin levels as well as with the maternal urine ghrelin levels. This statistical analysis was also performed taking into consideration the infants’ genders.

## 2. Results

Comparative characteristics of the study groups are presented in Tables S1 and S2. Data presented in Table 1 revealed that higher umbilical cord blood leptin and ghrelin concentrations were observed in the neonates born to the EGWG mothers. We compared the levels of leptin and ghrelin in all the tested materials depending on the sex of the newborns. Higher umbilical cord blood concentrations of leptin and ghrelin in the EGWG group were observed in the male neonates, while no such observations were made in the female infants. In the EGWG group, the mothers of the male newborns had significantly higher serum leptin levels (Table 1). 

We checked correlations between the neonatal anthropometric measurements and leptin and ghrelin levels. Taking into consideration the relatively small group of female infants in our study, we performed the correlation analysis only on male newborns in the EGWG and control groups. In the EGWG group the umbilical cord ghrelin levels correlated negatively with the neonatal birth weight and birth body length in all infants (Table 2) as well as in the male subjects (Table 3). Negative correlations were also found between the maternal urine ghrelin levels and neonatal birth weight and chest circumference in all studied infants in the EGWG group (Table 2), whereas in the male subjects only the chest circumference was negatively associated with the maternal urine ghrelin level (Table 3). The birth body length correlated positively with the umbilical cord leptin levels in the EGWG group (Table 2). This relation was not observed in the male subgroup (Table 3).

The controls presented umbilical cord ghrelin levels correlating positively with the neonatal birth weight, birth body length and head circumference (Table 4); whereas only with the head circumference measurements in the male subjects (Table 5). The maternal serum ghrelin levels correlated negatively with all these parameters but positively with the chest circumference in all control newborns (Table 4). In the male subgroup the maternal serum ghrelin level was negatively connected to the neonatal head circumference (Table 5). The urine ghrelin correlated negatively with all the anthropometric parameters of the neonates of the healthy group (Table 4), but in male subjects except for the head circumference measurements (Table 5). Positive correlations were found between the maternal serum leptin levels and all the neonatal anthropometric measurements except the head circumference, which correlated negatively with the umbilical cord leptin levels. These associations were statistically significant in all newborns in the control group (Table 4) as well as in the male subjects of this group (Table 5).

## 3. Discussion

Maternal pre-pregnancy BMI as well as gestational weight gain, connected to nutrition, have both independent and interacting effects not only on the fetal growth [4]. The majority of studies have investigated the relationship between the umbilical cord leptin and ghrelin and neonatal anthropometric measurements [9,16,17,18,19,20,21], however, their associations with the maternal serum and urine ghrelin concentrations are less studied. To the best of our knowledge, this study is the first to show the umbilical cord, maternal serum and urine ghrelin levels as well as the umbilical cord and maternal serum leptin levels in women with EGWG and the relationship of these parameters to the neonatal anthropometric measurements.

Our study showed that levels of leptin and ghrelin in the umbilical cord blood were statistically higher in male infants of the EGWG group in comparison to healthy subjects. We hypothesize that these differences can be connected to disparities in body composition and hydration status. Our previous study revealed that mothers with EGWG were characterized by increased fat and lean tissues in the bioelectrical impedance analysis [22]. Unfortunately, the evaluation of these parameters seems to not be feasible in the case of newborns.

Lecoutre et al. [23] observed a link between maternal obesity and adult rat offspring, where the latter were sensitized by the obese mother to increased visceral adiposity in a sex-specific manner. The cited authors demonstrated that maternal obesity programs visceral depots only in the male offspring in the group with a high-fat diet (HF; containing 60% lipids). Perirenal fat pads, yet not gonadal ones, were found to be involved in determining features in HF male offspring. On the basis of previous studies, the heterogeneity of the adipose lineage was proposed. It has been demonstrated that adipogenic stem cells and adipocytes act differently in the course of adipogenesis [24,25].

Previous studies revealed that females tended to have higher serum leptin levels than males [26]. Similar findings were observed in our study. Karakosta et al. [27] observed gender-specific differences in leptin levels in the umbilical cord blood of approximately 400 healthy neonates. The cited authors revealed that female subjects were characterized by higher levels of this adipokine than males [27]. It has also been observed that later in life the leptin levels are consistently higher in females in comparison to males [28,29].

Many authors are of the opinion that leptin concentration is higher in the mother than in the newborn [17,18,23,30]. Similar results were observed in the present study in all studied subjects and in groups of male infants. In both studied groups the leptin concentration was about 1.4 times higher in the blood of the mothers in comparison with the leptin concentration in the newborns. The discrepancy between leptin levels in the maternal serum and cord blood of male and female subjects can be connected to the phenomenon typical of this period of life when infants may be affected by an energy imbalance correlating with leptin levels [31]. Interestingly, a higher level of leptin was observed in the umbilical cord blood than in mothers in the group of female newborns. A similar dependence was described by Okdemir et al. [32] in the large for gestational age babies, however, these authors did not take into account the division of the group by sex of children.

Previous studies reported high ghrelin concentrations in the umbilical cord blood of pre-term and small-for-gestational-age infants. It was also reported that ghrelin levels were increased in the offspring of those women who had cigarette smoking habits and suffered from hypertension during pregnancy [33,34]. A limited and contrary amount of data is available on the umbilical cord ghrelin concentrations in the offspring of mothers with metabolic disturbances. Hehir et al. [35] did not detect a significant difference between healthy and type 1 diabetic pregnant women in this respect. However, Karakulak et al. [36] found that the umbilical cord blood ghrelin levels were decreased in the offspring of the gestational diabetes mellitus (GDM) women even after adjustment for birth weight, whereas Kara et al. [20] were able to notice similar ghrelin and leptin concentrations in the serum of the control and GDM mothers’ newborns. It has been suggested that ghrelin may play a role in the fetal adaptation to intrauterine malnutrition [34]. Our previous study detected lower serum and higher urine ghrelin concentrations in the early post-partum period when GDM women were compared with the health control group [37]. It is presumed that ghrelin in the cord blood is mainly secreted by the fetus. It is worth noting, however, that ghrelin is mainly secreted by the pancreas during the perinatal period rather than by the fundus of the stomach, which is typical of adult humans and rodents [38]. On the other hand, our findings of significant correlations between maternal serum and urine ghrelin levels and neonatal anthropomorphic measurements seem to be connected to the fact that a pregnant woman and her child may be perceived as a functional, complex unit. We decided to focus on urine as an easily obtainable biological material. Nonetheless, we are aware of the fact that small groups of participants represent a study limitation and we are not able to discuss these results in detail. Furthermore, it is not possible to relate our observations to the previously published papers of other authors since no such data exists.

There are many studies showing higher umbilical cord leptin levels in neonates born to obese mothers [17,21] but there are still limited data about leptin concentrations in women with EGWG. Previous studies concentrated on gestational weight gain in the first two trimesters of pregnancy, where EGWG was associated with higher levels of leptin and other parameters in the cord blood [8,39]. Allbrand et al. [21] observed increased level of the umbilical cord leptin and C-peptide in the infants born to obese versus normal-weight women. The present results confirm that the level of leptin in the umbilical cord blood was significantly higher in the EGWG patients than in the controls. These findings are similar to those reported by Biesiada et al. [17] who observed that leptin concentrations in the large for gestational age (LGA) children born to obese mothers were higher than in the LGA children born to mothers with normal BMI. 

It is worth highlighting that many of the above-quoted studies did not take into consideration the maternal BMI and weight gain during pregnancy. The results of our study revealed differences not only in the gestational weight gain between two groups of mothers but in the laboratory results as well. Lower levels of high-density lipoprotein cholesterol (HDL) as well as higher HgbA1c and triglycerides concentrations were present in the EGWG group. These results are consistent with the observations reported by other authors. Nonetheless, the offspring of the mothers from both studied groups presented similar anthropometric measurements, and their birth weight was also comparable. However, previous studies seem to confirm that the fetal metabolic programming may occur within normal birth weight ranges [40,41].

Researchers studying the relationship between ghrelin and anthropometric measurements revealed an inverse correlation between ghrelin concentrations and birth weight, height as well as BMI [42,43,44,45,46]. In their study, in which blood samples of diabetic mothers’ newborns were taken after birth before feeding, Kara et al. [20] found that only serum ghrelin negatively correlated with the birth weight. The authors concluded that this negative correlation of ghrelin, which seems to be a regulator of appetite, body fat mass, and energy balance, could potentially be advantageous to infants since appetite reduction might prevent postnatal excessive food intake and subsequent weight gain. Ding et al. [16] also suggested that ghrelin may play a role in regulation of body weight and energy homeostasis from the fetal period to adulthood. In our study the umbilical cord ghrelin levels negatively correlated with birth weight and birth body length in the EGWG group, they were also positively associated with the birth weight, birth body length, and head circumference in the control group. In the literature there is no clear evidence of consistent relationship between the ghrelin levels and neonatal anthropometry. There are also studies in which no correlation whatsoever has been reported [19,47,48,49]. 

The positive association detected in this study between the leptin concentration in the maternal serum and umbilical cord and neonatal anthropometric measurements confirms what was reported in the previous studies [9,17,30,50]. Schubring et al. [30] detected a significant correlation between leptin levels in the umbilical vein and artery and birth weight of the neonates. Biesiada at al. [17] observed moderate correlations between leptin concentrations and Ponderal Index, birth weight and the placenta weight. Samano et al. [9], similar to our findings in the control group, showed a correlation between the maternal leptin concentration and length of the newborn. This positive correlation between the cord blood leptin levels and birth weight could be indicative of leptin as a regulating factor responsible for fetal weight and its development. 

As other studies have reported, in our study the mean leptin concentrations were also higher in female neonates than in male neonates even though the birth weight in both sexes was similar. This may suggest participation of sex hormones in leptin secretion [17,50,51]. This is contrary to the study results published by Palcevska-Kocevsa et al. [50], who found no differences in the leptin concentrations between the male and female infants. In our study we observed significantly higher levels of leptin in the female neonates only in the control group. Similar levels of leptin between the males and females in the EGWG group may result from the fact that the male newborns had statistically higher levels of leptin in the umbilical cord blood in the group of patients with EGWGthan in the control group. Similar relationships, however, were not observed in the female newborns. 

Our results seem to highlight the importance of maternal condition, including gestational weight gain, in further research into the umbilical cord ghrelin and leptin concentrations and their relationship with the fetal and/or neonatal anthropometry and metabolic state.

The strength of this study lies in its novelty—the evaluation of the umbilical cord ghrelin levels of the EGWG mothers’ offspring as well as their associations with the maternal laboratory results and neonatal anthropometric measurements is an innovative approach. Nevertheless, the presented study has certain limitations. Firstly, it is indeed a relatively small-sample study. Secondly, we measured the levels of ghrelin and leptin in all the material only once. Therefore, it would be interesting to check how they change in time both in the mothers and in their offspring, which is quite motivating for us to continue our research into this issue and verify our results.

## 4. Materials and Methods

### 4.1. Study Population

The study consisted of Caucasian, singleton-term-pregnancy mothers (who completed 37 weeks of pregnancy) and infants delivered at the Chair and Department of Obstetrics and Perinatology, the medical University of Lublin, Poland. The study material was obtained between March 2016 and February 2017. The women were recruited at the time of delivery. We took into account only those in full-term pregnancy, i.e., after the completed 37th week of pregnancy calculated on the basis of the date of the last menstrual period or ultrasound examination in case the date of the last menstrual period was unknown. Two groups were selected on the basis of gestational weight gain: one group included women with normal gestational weight gain (11.5–16 kg; *n* = 28), while the other consisted of those with excessive gestational weight gain (≥20 kg; *n* = 38). Informed consent was obtained from each study subject and infant mother.

Characteristics of the study subjects also included: normal pre-pregnancy BMI values and three consecutive correct/normal results of 2 h-75 g-oral glucose tolerance test performed at 24–28 weeks of pregnancy [52,53], no concomitant diseases, and only vitamin-iron supplementation.

Exclusion criteria from the study included: multiple pregnancy, chronic infectious diseases, current urinary infections, abnormal laboratory results (e.g., the complete blood count, creatinine, glomerular filtration rate (GFR) findings); metabolic disorders (except improper gestational weight gain for the EGWG group), mental illness, cancer, liver diseases, cardiovascular disorders, fetal malformation, premature membrane rupture and intrauterine growth retardation.

The study protocol received approval of the Bioethics Committee of the Medical University of Lublin (no. KE-0254/221/2015 [25 June 2015] and no. KE-0254/348/2016 [15 December 2016]).

### 4.2. Measurements and Data Collection

Anthropometric measurements of mothers were performed shortly prior to and after delivery. Pre-pregnancy BMI values were determined during the first visits in the out-patients clinic, which were carried out up to 10 weeks of gestation. The following formula was used to calculate the gestational weight gain: the mother’s pre-pregnancy body mass subtracted from the weight at the day of delivery. We calculated gestational BMI gain as well. We defined gestational BMI loss as the difference between the mother’s BMI after delivery (during 48 h after delivery) and her BMI shortly prior to delivery. The newborn weights, lengths as well as the head and chest circumferences were measured right after delivery.

The cord blood samples’ collection was performed during delivery causing no hinderance to its course. The maternal serum and urine samples were taken after delivery, taking into account a 6 h fasting period. Samples were centrifuged at 1000× *g*, at 20 °C for 15 min. After centrifugation all the collected cord blood serum as well as the serum and urine samples obtained from the studied mothers were stored at −80 °C. Ghrelin concentrations were determined with the use of kits available on the market and in agreement with the manufacturer’s instructions (Wuhan EIAab Science Co., Wuhan, China) via traditional enzyme-linked immunosorbent assay (ELISA). Detection range for ghrelin was 0.156–10 ng/mL. Concentrations of leptin in cord blood serum and maternal serum and urine were measured by means of commercially available kits and in agreement with the manufacturer’s instructions (R&D Systems, Inc., Minneapolis, MN, USA) via ELISA. The threshold of leptin sensitivity was equal to 7.8 pg/mL, while the reference range for women was 3877–77,273 pg/mL. Since the leptin urine levels in the majority of the studied subjects were below the threshold of sensitivity of the ELISA test, the “urine leptin” parameter was not included in our study results. The measurements of maternal serum levels of albumin, fasting blood glucose (FBG), hemoglobin A1c (HgbA1c) as well as the lipid profile were performed by an authorized laboratory. 

This is the second analysis based on subjects from the control group that was previously used in our study [37]. In the cited study we compared women diagnosed with gestational diabetes mellitus (GDM) with the aforementioned control group [37]. The cohort analyzed in the present study was used to measure SFRP5 and was described in our previous study [22]. Comparative characteristics of the study groups are presented in Tables S1 and S2.

### 4.3. Statistical Analysis

All the obtained results are presented as the median (interquartile range 25–75%) or as numbers and percentages. Differential significance test was conducted by means of Mann–Whitney U test. Correlation analyses used Spearman’s coefficient test and were performed with the use of Statistical Package for the Social Sciences software (version 19; SPSS Inc., Chicago, IL, USA); *p* < 0.005 was assumed to be statistically significant. 

## 5. Conclusions

Our study revealed that the umbilical cord leptin and ghrelin levels were significantly higher in the offspring of the EGWG mothers, but only in the male newborns. Our study results indicate that the condition of the mother, i.e., her BMI values both in the periconceptional and periparturient periods as well as her metabolic parameters (e.g., her lipid profile), may affect her offspring’s ghrelin and leptin concentrations at delivery. 

Differences in the correlations between the leptin and ghrelin concentrations (both in the umbilical cord blood and maternal serum as well as in urine) and the anthropometric results of the neonates are dependent on the studied group. This can be exemplified by the umbilical cord ghrelin which correlates negatively with the birth weight and birth body length in the EGWG group, while its correlation with the birth weight, birth body length and head circumference is positive in the control group. These significant correlation differences result from different levels of ghrelin in the umbilical cord blood (significantly higher level in the EGWG group, at *p* < 0.0001) with comparable birth measurements in both studied groups. However, the possibility of fetal metabolic programming occurrence within the normal birth weight ranges, as has previously been reported by other authors [40,41], should be stressed once more. 

## Figures and Tables

**Table 1:** Comparison of the study groups.

Variables	EGWG Group (*n* = 38)	Control Group (*n* = 28)	*p*
Male infant, *n* (%)	24 (63.2)	20 (71.4)	0.66
Female infant, *n* (%)	14 (36.8)	8 (28.6)	0.66
Cord blood ghrelin, ng/mL	0.52 (0.26–1.83)	0.19 (0.19–0.28)	**0.00001**
Male	0.49 (0.27–0.8)	0.19 (0.19–0.2)	**0.0001**
Female	0.56 (0.25–20.11)	0.28 (0.23–0.29)	0.44
Cord blood leptin, ng/mL	10.99 (8.5–13.4)	7.53 (4.9–14.01)	**0.00003**
Male	10.8 (8.7–12.6)	7.3 (4.9–7.8)	**0.03**
Female	21 (8.2–22.3)	14 (11.7–14.6)	0.36
Maternal serum ghrelin, ng/mL	1.19 (0.34–2.43)	0.93 (0.65–1.12)	0.63
Male	1.1 (0.44–1.73)	0.97 (0.9–1.1)	0.42
Female	2.7 (0.2–16.7)	0.25 (0.21–0.39)	0.34
Maternal urine ghrelin, ng/mL	0.12 (0.04–0.3)	0.1 (0.1–0.29)	0.75
Male	0.25 (0.05–0.34)	0.1 (0.1–0.29)	0.92
Female	0.08 (0.03–0.12)	0.1 (0.1–0.25)	0.26
Maternal serum leptin, ng/mL	14.87 (12.6–47.6)	10.43 (6.04–14.9)	0.06
Male	15 (14.6–61.7)	14.6 (6.04–14.9)	**0.01**
Female	12.3 (11.8–33.4)	6.5 (6.3–7.3)	0.09

The results are shown as the median (interquartile range 25–75%). Statistically significant values are given in bold. EGWG—Excessive gestational weight gain.

**Table 2:** Correlations of neonatal anthropometric measurements in the EGWG group (Spearman’s rho coefficient).

Variables	Birth Weight	Birth Body Length	Head Circumference	Chest Circumference
Umbilical cord ghrelin level	−0.560 *	−0.727 ***	−0.203	−0.331
Maternal serum ghrelin level	-0.105	−0.309	−0.170	0.001
Maternal urine ghrelin level	−0.452 *	−0.320	−0.174	−0.596 *
Umbilical cord leptin level	0.326	0.572 *	0.063	−0.044
Maternal serum leptin level	−0.151	−0.133	0.066	−0.228

Statistically significant values are given in bold. * *p* < 0.05; *** *p* < 0.0001; EGWG—excessive gestational weight gain.

**Table 3:** Correlations of neonatal anthropometric measurements in the EGWG group in male subjects (Spearman’s rho coefficient).

Variables	Birth weight	Birth Body Length	Head Circumference	Chest Circumference
Umbilical cord ghrelin level	−0.733 **	−0.829 ***	−0.271	−0.747 **
Maternal serum ghrelin level	−0.300	−0.393	−0.140	−0.378
Maternal urine ghrelin level	−0.450	−0.419	−0.420	−0.615 *
Umbilical cord leptin level	0.445	0.445	0.437	0.413
Maternal serum leptin level	−0.133	−0.265	0.061	−0.351

Statistically significant values are given in bold. * *p* < 0.05; ** *p* < 0.001; *** *p* < 0.0001; EGWG—excessive gestational weight gain.

**Table 4:** Correlations of neonatal anthropometric measurements in the control group (Spearman’s rho coefficient).

Variables	Birth Weight	Birth Body Length	Head Circumference	Chest Circumference
Umbilical cord ghrelin level	0.486 *	0.525 *	0.794 ***	−0.278
Maternal serum ghrelin level	−0.543 *	−0.617 *	−0.706 **	0.432 *
Maternal urine ghrelin level	−0.771 ***	−0.833 ***	−0.441 *	−0.463 *
Umbilical cord leptin level	−0.286	−0.092	−0.559 *	0.061
Maternal serum leptin level	0.600 *	0.494 *	0.294	0.833 ***

Statistically significant values are given in bold. * *p* < 0.05; ** *p* < 0.001; *** *p* < 0.0001.

**Table 5:** Correlations of neonatal anthropometric measurements in the control group in male subjects (Spearman’s rho coefficient).

Variables	Birth Weight	Birth Body Length	Head Circumference	Chest Circumference
Umbilical cord ghrelin level	0.200	0.289	0.669 *	−0.335
Maternal serum ghrelin level	−0.200	−0.289	−0.667 *	0.335
Maternal urine ghrelin level	−0.700 **	−0.866 ***	−0.205	−0.671 **
Umbilical cord leptin level	−0.500 *	−0.577 *	−0.820 ***	0.112
Maternal serum leptin level	0.900 ***	0.866 ***	0.410	0.783 ***

Statistically significant values are given in bold. * *p* < 0.05; ** *p* < 0.001; *** *p* < 0.0001.

## Supplementary Materials

Supplementary materials can be found at https://www.mdpi.com/1422-0067/20/10/2398/s1.
