# Supplementary material for: Leptin and Ghrelin in Excessive Gestational Weight Gain—Association between Mothers and Offspring
Source: Int J Mol Sci. 2019 May 15;20(10):2398. doi: 10.3390/ijms20102398 (PMC6566238; doi:10.3390/ijms20102398)
Supplement: Supplementary file 1 [file ijms-20-02398-s001.pdf]

Table 1. Comparison of the demographic and laboratory characteristics of the subjects.

Variables	EGWG group (n = 38)	Control group (n = 28)	p
Age, years	29 (28–32)	29 (24–38)	0.84
Pre-pregnancy BMI, kg/m^2	23.2 (21.6–24.09)	20.3 (19.5–24.4)	0.06
Gestational weight gain, kg	23.9 (21–26)	15 (11.5–15.6)	**0.00001**
BMI before delivery, kg/m^2	31.3 (29.7–32.05)	26.3 (24.2–29.1)	**0.00001**
BMI after delivery, kg/m^2	28.6 (26.2–29.7)	22 (21–23.9)	**0.00001**
ΔBMI 1, kg/m^2	8.4 (7.07–9.4)	5.4 (3.0–5.6)	**0.00001**
ΔBMI 2, kg/m^2	2.75 (2–3.2)	2.5 (2.08–4.16)	0.88
Cesarean percent (%)	26	14	0.38
FBG, mg/dL	80.55 (78–86)	83.5 (73–91)	0.55
Albumin, g/dL	3.55 (3.41–3.81)	3.68 (3.43–3.73)	0.68
Total cholesterol, mg/dL	225 (197–249)	249 (188–287)	0.64
HDL, mg/dL	71 (59–79)	78 (75–82)	**0.03**
LDL, mg/dL	106 (87–128)	129 (93–152)	0.23
Triglycerides, mg/dL	204 (178–258)	177 (150–254)	**0.01**
HgbA1c, %	5.5 (5.0–5.5)	5.3 (4.6–5.4)	**0.03**

The results are shown as the median (interquartile range 25%–75%). Statistically significant values are given in bold. BMI—body mass index; **Δ** BMI 1—gestational BMI gain; **Δ** BMI 2—BMI loss after delivery; EGWG—Excessive gestational weight gain; FBG—fasting blood glucose; HDL—high-density lipoprotein cholesterol; LDL—low-density lipoprotein cholesterol; HgbA1c—hemoglobin A1c.

Table 2. Comparison of the neonatal characteristics of the study subjects.

Variables	EGWG group (n = 38)	Control group (n = 28)	p Value
Birth weight, g	3520 (3400–3650)	3630 (3200–3920)	0.88
Birth body length, cm	55 (54–56)	56 (55–57)	0.08
Head circumference, cm	34 (33–35)	34 (33–35)	0.59
Chest circumference, cm	34 (33–35)	34 (34–35)	0.08

The results are shown as the median (interquartile range 25–75%). EGWG—Excessive gestational weight gain.
